# Enhancing free choice masked priming *via* switch trials during repeated practice

**DOI:** 10.3389/fpsyg.2022.927234

**Published:** 2022-09-08

**Authors:** Qi Dai, Lichang Yao, Qiong Wu, Yiyang Yu, Wen Li, Jiajia Yang, Satoshi Takahashi, Yoshimichi Ejima, Jinglong Wu

**Affiliations:** ^1^Cognitive Neuroscience Laboratory, Graduate School of Interdisciplinary Science and Engineering in Health Systems, Okayama University, Okayama, Japan; ^2^School of Education, Suzhou University of Science and Technology, Suzhou, China; ^3^Beijing Institute of Technology, Beijing, China

**Keywords:** masked priming effect, free choice, subliminal learning effect, masked stimulus processing, intention

## Abstract

The masked priming paradigm has been extensively used to investigate the indirect impacts of unconscious stimuli on conscious behaviors, and the congruency effect of priming on free choices has gained increasing attention. Free choices allow participants to voluntarily choose a response from multiple options during each trial. While repeated practice is known to increase priming effects in subliminal visual tasks, whether practice increases the priming effect of free choices in the masked priming paradigm is unclear. And it is also not clear how the proportions of free choice and forced choice trials in one block will affect the free choice masked priming effect. The present study applied repeated practice in the masked priming paradigm and found that after training, the participants were more likely to be influenced by masked primes during free choice, but this training process did not alter the visibility of masked stimuli. In addition, this study revealed that when the proportions of free choice and forced choice trials were equal during the training stage, this enhanced effect by practice was the strongest. These results indicated that practice could enhance masked stimulus processing in free-choice, and that the learning effect may mainly be derived from the early selection and integrated processing of masked stimuli.

## Introduction

Making a choice is a complex process. Every day, people face an uncountable number of choices. The ability to make such voluntary, or free, choices is fundamental to being a human. To fulfill our goals or desires, we constantly interact with the external environment through our voluntary behaviors. In more recent years, many studies aimed to uncover the functional neuroanatomy of free choices, typically by comparing free choices with forced choices (Orr and Banich, 2014; Teuchies et al., 2016). In this field of research, free choice and forced choice are defined as stimulus-based and stimulus-independent choices, respectively (Zhang et al., 2012; Wisniewski et al., 2016). Furthermore, in the free choice paradigm research, participants make a voluntary choice from multiple options during each trial. The available options can be similar to each other. Importantly, the participants are aware that all available options are homogeneous in terms of their objective outcomes, and that the tasks do not introduce or manipulate rewards or costs according to the choices made. Therefore, the task is not to identify a correct response (Si et al., 2021). These studies commonly use variants of the “free choice” paradigm to determine the neurocognitive mechanisms of voluntary decision processes (Dall’Acqua et al., 2018; Si et al., 2021).

Priming is a phenomenon by which exposure to one stimulus (as a prime) influences how a person responds to a subsequent, related stimulus (as a target; Henson et al., 2014). Recent studies have demonstrated that masked primes can affect the motor system, thus affecting the free choice. Although primes below the identification threshold have been presented, the reaction times and accuracy differed depending on whether prime–target relationships were congruent or not (Lingnau and Vorberg, 2005; Wenke et al., 2010; Chambon et al., 2013). In a free choice masked priming task, participants are more likely to choose the target congruent with the prime than the target incongruent with the prime. The reaction time is shorter, and the error rate is lower when the congruent target is chosen in a forced choice (Mattler and Palmer, 2012). Schlagheken and Eimer’s study (Schlaghecken and Eimer, 2004) focused on how masked primes affected free choice. This study included blocks in which the participants always responded with free choices (free: forced = 100:0) and blocks in which free and forced choice responses were mixed (free: forced = 50:50). When free choice responses were mixed with forced choice responses, the responses were susceptible to the priming effect. However, when free choices responses were performed alone, the priming effect was not observed. This result suggests that a combination of forced choices and free choices are necessary for masked priming effect to occur with free choices; only primes that are a part of the current active task set, i.e., the set of stimulus–response (S–R) mappings imposed by the task instructions and applied by the participants, affect the motor system. Mattler and Palmer (2012) used different stimulus sets in different experiments, while the responses were defined spatially (left vs. right). In experiment 1, the stimuli were arrows (pointing left vs. right). In experiment 2, the stimuli were arbitrary shapes (square vs. diamond). In experiment 3, the authors used physical locations (left vs. right). The authors found a significant priming effect on free choice responses when the stimuli provided explicit spatial information (arrows and physical location). When the stimuli were arbitrary shapes and not readily grouped with response locations, the primes were relatively ineffective in biasing the free choice responses. The authors indicated that the reason is that these stimuli are more easily grouped with responses. Because for the responses of left or right, stimuli with clear spatial information are more strongly associated with responses. Therefore, the masked priming phenomenon of free choice suggests that invisible information can influence free choices and that stronger S-R mapping in the forced choice task can increase the susceptibility of free choices to a priming effect.

Some studies have shown that repeated practice improves performance in a wide variety of visual tasks; For example, the ability to discriminate between visual stimuli is improved after training (Saarinen and Levi, 1995). The above process can occur while processing a subliminal stimulus, e.g., a masked prime, which is called subliminal learning (Seitz and Watanabe, 2003). Vlassova and Pearson used a moving point as a mask to convert stimuli into subliminal stimuli and performed repeated practice to investigate the effect of subliminal learning (Vlassova and Pearson, 2018). These authors found that the achieved reaction accuracy was significantly higher than that yielded in the no-training task, suggesting that the participants could improve their choice performance through training. Moreover, since different stimulus moving directions are used in the test task and the training task, the results indicated that the subliminal learning effect is not based on increases in task familiarity but is based on the change in information selection and integration (Vlassova and Pearson, 2018). Although, several studies have also demonstrated perceptual learning of unconscious, masked stimuli (Schwiedrzik et al., 2009, 2011), these learning effects were shown to be limited to lower levels of the visual processes as the performance increase did not transfer to untrained spatial locations. In Vlassova and Pearson’s study, different stimulus moving directions were used in the test task and the training task, and the results indicated that the subliminal learning effect was not based on increases in low-level motion or vision processes but was based on the change in information selection and integration (Vlassova and Pearson, 2018). In addition, identification accuracy in a partially masked stimulus improved following training using a completely masked stimulus. The authors fit each participant’s data using the drift diffusion model and find that the model is best explained when the drift rate is allowed to vary. The drift rate is defined in the drift diffusion model as the average rate of information accumulation. The higher the drift rate, the faster and more accurate the response. This result suggested that the sensory evidence was unconsciously accumulated improved following a period of training on a stimulus in the complete absence of perceptual awareness. That is, processing of masked stimuli improves with repeated practice, but the increase showed only when decision-relevant conscious information correlated to the masked information is present. When the masked information is not related to decision-relevant conscious information, the learning effect does not occur (Vlassova and Pearson, 2018). Interestingly, in the free choice masked priming, the masked prime is not directly related to the free choice target stimulus (decision-relevant conscious information), therefore, whether repeated practice can produce subliminal learning effects is unclear.

Furthermore, when both free choice and forced choice tasks are present in a block, it is not clear whether repeat trials or switch trials between the free choices and forced choices are more important for inducing free choice masked priming. Some researchers have indicated that participants are more susceptible when switching between different tasks (Dreisbach and Fröber, 2019). In addition, some studies have shown that when switching between the free choice and the forced choice types, the response characteristics are still maintained, and the two tasks can share S-R mapping (Hughes et al., 2011). Therefore, compared to repeat trials, switch trials should have a stronger priming effect on free choice responses. However, it has also been indicated that repeat trials can reinforce S-R mapping compared to switch trials (Dreisbach and Fröber, 2019). If the initiation of free choice responses depends on the strength of the stimulus–response association in the forced choice task, then, compared with switch trials, repeated trials should have a stronger priming effect on the free choices (Gozli, 2019). However, this problem is difficult to solve by comparing different conditions in priming experiments because different repeat trials inevitably lead to different numbers of valid trials, which greatly affects the reliability of the statistics. Therefore, in the present study, we investigated the learning effect by training free choice priming with different numbers of switch trials and repeat trials and speculate which of the two has a greater impact on free choice masked priming.

The present study has the following two research aims: (1) this study aims to determine whether the subliminal learning effect can be produced in masked priming free choices after repeated practice, and (2) three training conditions with different ratios of free choices and forced choices in training session were selected to represent higher switch trials (free choice: forced choice = 50:50) and higher repeat trials (free choice: forced choice = 10:90 and 90:10) to determine the learning effect through practice and find out which of the switch trials and repeat trials has a greater impact on the priming effect. This study included four experiments. In Experiment 1, the participants were asked to train 4 times, and the intensity of the priming effect of free choice before and after training was calculated to determine whether the subliminal learning effect appears in the free choice trials. Furthermore, through changing the location and the direction indicated by the stimuli during the training phase (training task) and the testing phase (pre- and post-test task), we can determine whether the subliminal learning effect produces cognitive changes by comparing pre-test and post-test results. In Experiment 2, training unrelated to the subliminal priming effect was conducted as a control experiment to ensure that the subliminal learning effect was derived from the training process. In Experiments 3 and 4, we changed the proportion of the free choice trials and forced choice trials in each block during the training task. In Experiment 3, each training block included 10% free choices trials and 90% forced choice trials. In Experiment 4, each training block included 90% free choices trials and 10% forced choice trials. We calculated the intensity of the priming effect on free choice before and after the training and compared it with the results of the control group in Experiment 1 to determine whether the proportions of free choices and forced choices in a block modulated the subliminal learning effect.

## Materials and methods

### Participants

A statistical power analysis was performed for the sample size estimation. A 2 (pre-test vs. post-test) × 2 (groups: Experiment 1 vs. Experiment 2) mixed design and a 2 (pre-test vs. post-test) × 3 (groups: Experiments 1 vs. 3 vs. 4) mixed design were used in our experiments to calculate the sample size. Accordingly, the configuration parameters in G*Power version 3.1 (Faul et al., 2007) were used. The projected partial *η*^2^ of the interaction between the group factor and pre-post factor was set at 0.25, which is considered stricter than the *a priori* results of similarly designed experiments conducted by Mattler and Palmer (2012). The two-tailed alpha level was set at 0.05; the power value was set at 0.90; the number of groups was set at 2; and the number of measurements was set at 4. Therefore, a sample size of 15 was required per group. Therefore, we finally recruited a total of 60 participants, which was adequate for the main objective of this study. Fifteen participants were recruited for each experiment (Experiment 1: 7 males, 22–31 years old; Experiment 2: 7 males, 22–34 years old; Experiment 3: 7 males, 23–30 years old; and Experiment 4: 5 males, 22–26 years old), for a total of 60 participants for all 4 experiments. All participants reported normal or corrected-to-normal vision and provided written informed consent, which was previously approved by the ethics committee of Okayama University and was conducted in accordance with the Declaration of Helsinki.

### Apparatus and stimuli

MATLAB software (R2014b, MathWorks, MA, Psychtoolbox, 3) was used to present the stimuli and record the experimental data. In a dark room, a display (144 Hz, 24 inch, iiyama ProLite GB2488HSU-B1) with a refresh rate of 120 Hz and an observation distance of 60 cm was used to perform the task.

**Figure 1:**
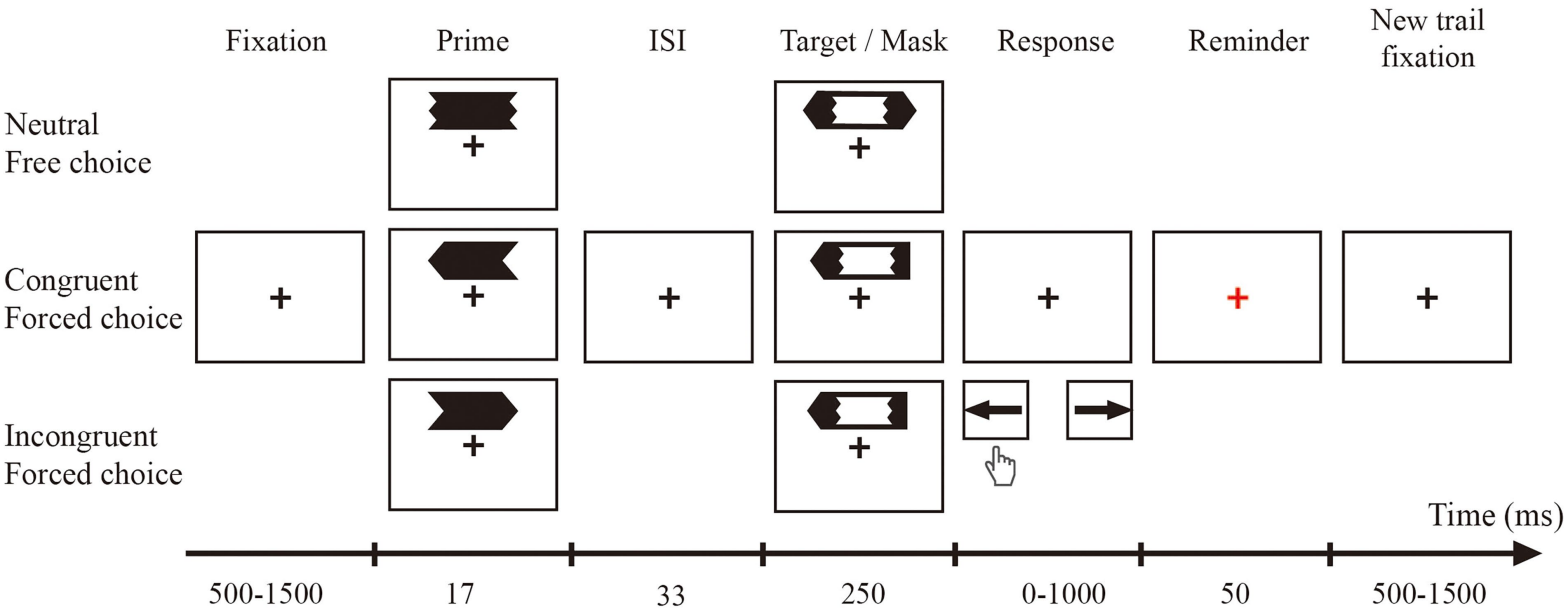
Schematic of the trial procedures and stimuli in the pre-and post-test tasks. Three example trials of the possible combinations showing the choice type (free: upper row; forced: middle and lower row) and prime target congruency (neutral: upper panel; congruent: middle panel; incongruent: lower panel). The participants were instructed to respond to the target stimuli while being unaware of the presence of the arrow primes. Primes and targets could appear randomly above or below their fixation point during each trial, and the orientation of each stimulus was horizontal. In the illustrations in the article, the background is shown as white, and the stimuli are shown in black to enhance legibility. However, in the actual experiment, the background was black, and the stimuli were white.

**Figure 2:**
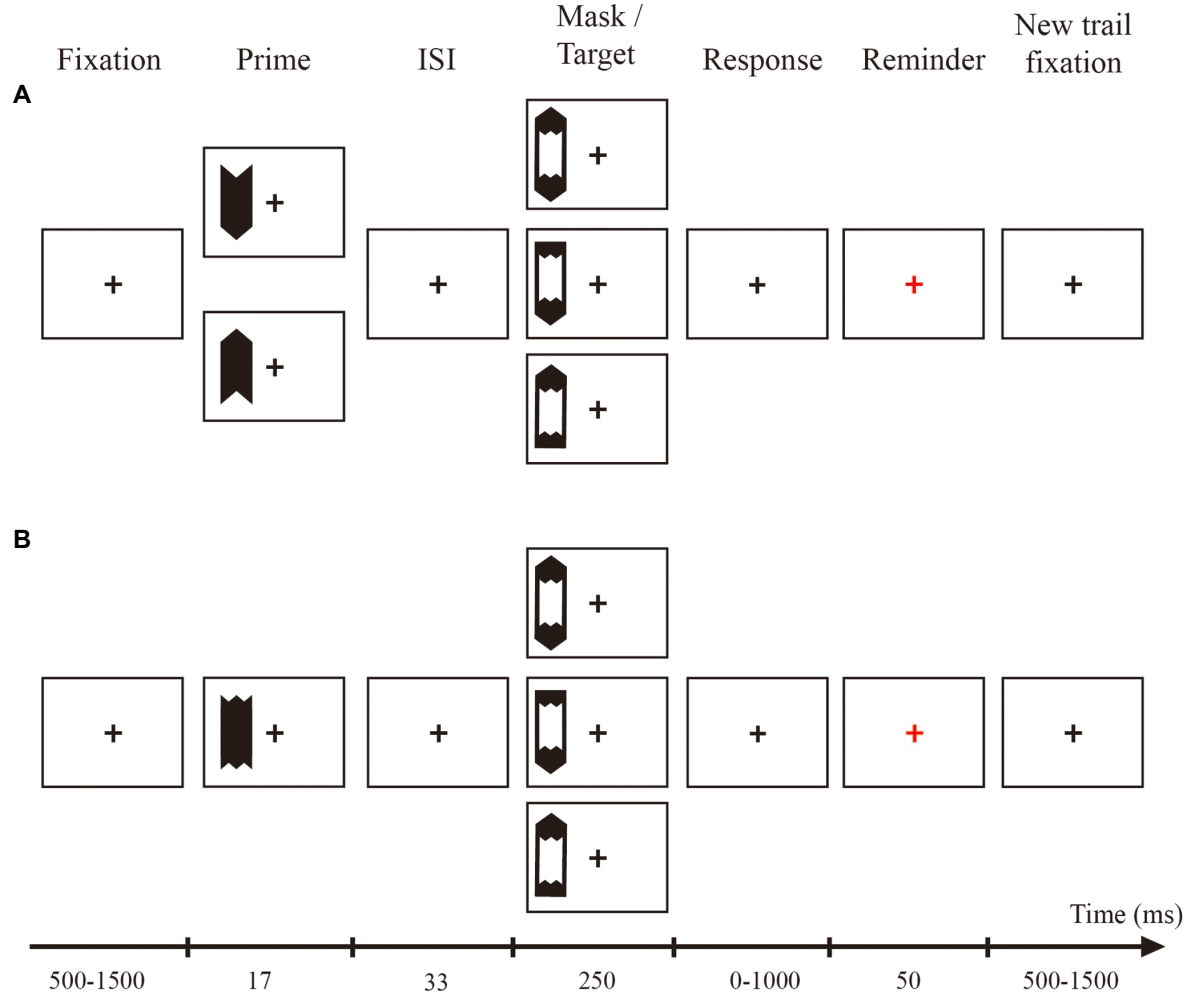
Schematic of the trial procedure and stimuli in the training task. During the training phase, the presentation position of the prime and target was 1.38° to the left or right of the fixation point, and the arrow pointing was up or down. The size and shape of the prime and target were always the same. Panel **(A)** shows the specific sequence of each trial during the training phases of Experiments 1, 3 and 4. In these three experiments, the prime always pointed up or down. Panel **(B)** shows the specific sequence of each trial during the training phase of Experiment 2 (control experiment). In Experiment 2, the prime arrow pointing direction was always “no direction.”

The priming task was adapted from a study by Mattler and Palmer (2012). We used the following three types of prime stimuli: a white left-and right-pointing arrow and a nondirectional stimulus (which consisted of overlapping left-and right-pointing arrows). The primes were followed by metacontrast masks of the same luminance. Metacontrast masking refers to the suppression of the visibility of a briefly flashed target stimulus by a similarly brief and spatially adjacent mask stimulus that follows the target in time at varying stimulus onset asynchronies (SOAs; Breitmeyer et al., 2008). The metacontrast mask was embedded in the target stimulus, pointing to the left or right in the forced choice task or in both directions in the free choice task. The visual angle of the prime was 0.8° × 1.86°, and the target visual angle was 1.09 × 3.47°. As shown in Figure 1, in the test, the prime stimulus and target stimulus could randomly appear above or below the fixed cross at a viewing angle of 1.38°. The unpredictable location was reported to enhance the masking effect (Vorberg et al., 2003). Previous studies have also found that such experimental parameters can ensure that the primes are always below the identification threshold (Teuchies et al., 2016). In addition, this study measured the visibility of the prime and found its at chance level. As shown in Figure 2, in the training, the prime stimulus and target stimulus could randomly appear on the left or right side of the fixed cross at a viewing angle of 1.38°. The pointing direction of the arrows changed from vertical (pointing up and down) to horizontal (pointing left and right) during the testing phase.

### Procedure

During the test phase (pre-test and post-test), the participants had to make two different responses: free choice and forced choice. When a two-direction arrow (free choice target arrow, see Figure 1) appeared on the screen, the participants were allowed to choose freely. Under the free choice condition, the participants were free to respond left or right by pressing a button. Additionally, the participants were encouraged to perform each response with equal frequency rather than make a fixed response. When a one-direction arrow appeared on the screen, the participants needed to choose the appropriate button in the indicated direction. An example of each target stimulus was shown to the participants before the experiment to ensure that the participants were familiar with the target. As shown in Figure 1, the presentation time of the prime was 17 ms, followed by an interstimulus interval (ISI) of 33 ms at the beginning of the target stimulus. The target duration was 250 ms. The reaction window was set to 1,000 ms. Once a participant pressed the button, the fixation point briefly turned red to remind the participant. Notably, the reminder was only used to inform the participants that the button was pressed and not whether the response was correct. If a participant did not respond during this time window, the program skipped to the next step. Under the forced choice condition, the target arrow pointed to the left half of the time and to the right half of the time. These three primes were also presented randomly, and the probability of presenting each prime stimulus was equal. The prime stimulus and target stimulus were always presented in the same positions. The test session consisted of three blocks, each with 180 trials. We recorded the response made by the participant and the reaction time from the appearance of the target stimulus to the participant pressing the button. When a one-direction arrow appeared on the screen, the participants needed to choose the appropriate button representing the indicated direction.

In the pre/post-test of the present study (see Figure 1), the position of the prime stimulus and the target stimulus was directly above or below the fixation point, and the target arrow pointed to the left and right. The participants pressed the left key and right key to respond to the target. During the training phase (see Figure 2), the position of the prime stimulus and the target stimulus was directly on the left or right side of the fixation, and the target arrow pointed up and down. The stimulus size and time course were the same as those in the test phase. When a free choice target arrow appeared on the screen, the participants were allowed to choose freely. Under the free choice condition, the participants were free to choose up or down by pressing a button.

Similar to numerous previous studies, we classified all responses of the participants as congruent and incongruent. Under the forced choice condition, in the congruent trials, the direction of the prime was the same as that of the response that indicated the direction of the target arrow. And in the incongruent trials, the response was again the same as the target direction but opposite to the prime direction. In the free choice trials, congruency was defined by the relationship between the response of the participants and the prime because there were no correct target responses in these trials. When the participants “freely” chose a reaction in the same direction as the prime, the reaction was considered congruent, and when their reaction was opposite to the direction of the prime, the reaction was defined as incongruent.

### Prime visibility test

Following the test phase, participants were explicitly told about the presence primes, and performed a prime visibility test. This test allowed us to check if the prime stimuli were indeed presented subliminally, or not. Several researchers have indicated that the prime visibility test must be identical to the main priming task because differing task demands can modulate the visibility measure (Eriksen, 1960). Therefore, the prime visibility test experimental procedure used the exact same procedure as the test phase, that is, a prime stimulus was presented first with a duration of 17 ms, followed by an interstimulus interval (ISI) of 33 ms at the beginning of the target stimulus. The target duration is 250 ms. Participants were only required to identify the direction of the prime pointing (left, right or no direction) on each individual trial by using the 1, 2, 3 button in the keyboard and ignore the target stimulation. The prime visibility test was performed after the end of the test experiment on the first and last day of the experiment, and each visibility test block included 60 trials.

### Experimental design

**Figure 3:**
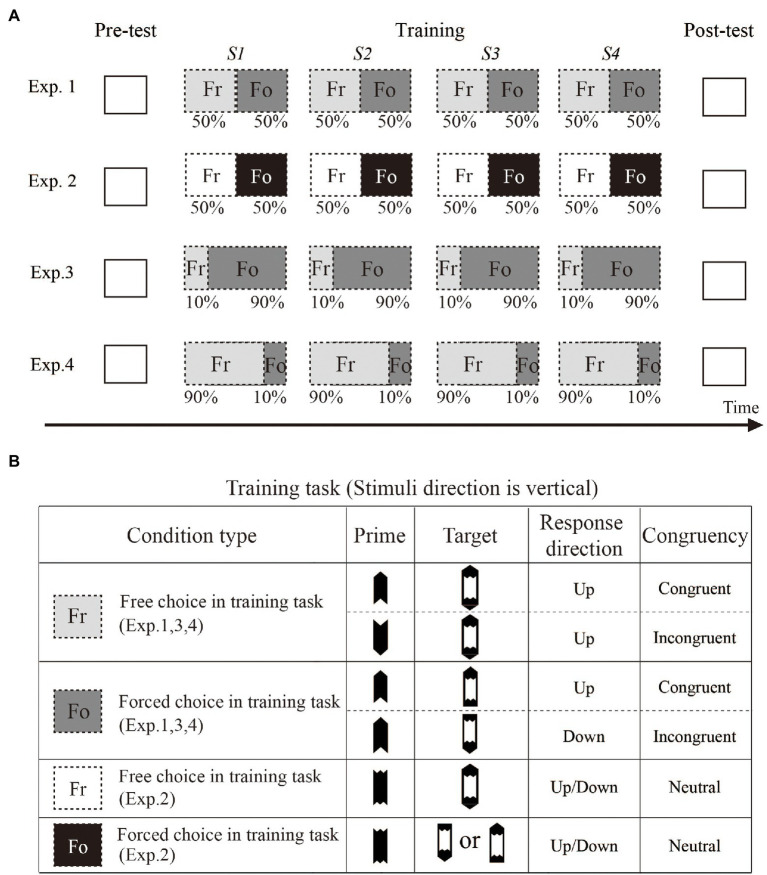
**(A)** shows schematic outlining of the test and training sessions of Experiments 1–4. During the pre-and post-test phases, the participants needed to complete one test session (three blocks). During the training phase, the participants completed four training sessions (three blocks per session). The interval between each pair of sessions was at least 6 h. In Experiments 1–4, the test trials were the same, and only the training sessions were manipulated. Panel **(B)** shows the stimulus type and definition of congruency in the training task. Notably, the direction of the prime during the training phase of Experiment 2 was always neutral. In the other 3 experiments, the prime direction was pointed always vertically.

Experiment 1 included a training phase and a pre-test/post-test phase (see Figure 3). For all participants, the experimental stimulus and time course of the test task were the same. In each block during the test phase, there were 50% free choices (90 trials) and 50% forced choices (90 trials). Regarding the time interval, we used a time interval design similar to that used in Vlassova and Pearson (2018) to ensure a stable learning effect. During the training phase, four training sessions were completed in 3 days, and at least 6 h lapsed between sessions. The participants completed four training sessions; each session included 3 blocks, and each block included 160 trials. In Experiment 1, the proportions of free choice trials and forced choice trials in one block were equal. Therefore, each training block included 80 free choice trials and 80 forced choice trials randomly interspersed.

In Experiment 2, the experimental tasks and stimuli during the pre/post-test phase were the same as those in Experiment 1; however, during the training phase, although 50% of the free choice and forced choice trials in each block were the same as those in Experiment 1, there was no directional prime in each block. Compare to Experiment 1, although the participants in Experiment 2 were trained 4 times as the control group, with this method, it was difficult for the masked-stimulus directional information to affect their responses during the training process.

In Experiments 3 and 4, the experimental tasks and stimuli during the pre/post-test phase were exactly the same as those in Experiment 1. To determine whether the ratio of free choice to forced choice trials during the training phase had an impact on the learning effect, the free choice and forced choice trial proportions in each training block during the training stage were changed. Experiment 3 consisted of 10% free choice trials and 90% forced choice trials; thus, there were 16 free choice trials and 144 forced choice trials in a training block. Experiment 4 consisted of 90% free choice trials and 10% forced choice trials; thus, there were 16 forced choice trials and 144 free choice trials in a training block. By calculating the intensity of the priming effect of the free choice trials before and after training and comparing it with the control group, we can determine whether the influence of the ratio of free choice to forced choice trials affected the learning effect.

### Analyses of data

We deleted all data with response times less than 100 ms or greater than 1,000 ms because these data were considered abnormal data. We only calculated the average reaction time of all correct trials. To confirm the priming effect in each group, the average reaction time and the error rate in the pre-test and post-test were measured. The average reaction time was analyzed using a 2 (pre-test and post-test) × 3 (congruent, incongruent and neutral) repeated analysis of variance. Under the free-choice condition, the congruent response rate was calculated. By comparing the congruent response rate in the pre-test and post-test, the congruent response rates in the pre-test and post-test of Experiment 1 and Experiment 2 were analyzed by a 2 (pre-test and post-test) × 2 (groups: Experiment 1 vs. Experiment 2) mixed analysis of variance to determine whether subliminal learning enhanced the subliminal priming effect. The congruent response rates in the pre-test and post-test of Experiments 1, 3 and 4 were analyzed by a 2 (pre-test and post-test) × 3 (groups: Experiments 1 vs. 3 vs. 4) mixed analysis of variance to determine whether the proportion of free choice and forced choice trials during training impacted the learning effect. We also performed a repeated-measures analysis of variance according to the differences in the congruent response rate before and after training (post-pre) in the four experiments. In addition, to determine whether the visual abilities of the participants changed, we used an independent-samples t-test to calculate the prime visibility rates before and after the training. To evaluate whether the participants in different experiments had different changes in prime visibility, the prime visibility test results produced before and after training in the 4 experiments were submitted to a 4 (groups: Experiments 1 vs. 2 vs. 3 vs. 4) × 2 (pre prime visibility test vs. post prime visibility test) mixed analysis of variance.

## Results

First, a one-samples t-test was used to determine whether there was a difference between the mean correct prime recognition percentage of all participants and the chance level (0.33). The data had no significant outliers and were close to a normal distribution. The results showed that the participants’ percentage of correct prime recognition was 0.332 ± 0.043 before training, and the difference from the chance level was 0.002 (95% confidence interval-0.010 ~ 0.013). The one-samples t-test results indicated that the difference was not significant (*t*(59) = 0.297, *p* = 0.767). The participants’ percentage of correct prime recognition was 0.325 ± 0.055 after training, and the difference from the chance level was –0.005 (95% confidence interval --0.019 ~ 0.009). The one-samples t-test results indicated that that the difference was not significant (*t*(59) = −0.699, *p* = 0.487). Thus, there was no significant difference between the prime visibility and the chance level both before and after training. This result indicated that the primes were not consciously discernible to the participants. In addition, both before and after the training, prime visibility was submitted to a paired t-test, which indicated that the correct recognition percentage was not significantly enhanced by the training (post–pre = −0.007, *t*(59) = 0.700, *p* = 0.487). The results of the 4 (Experiments 1, 2, 3 and 4) × 2 (pre prime visibility test vs. post prime visibility test) mixed ANOVA also showed that the participants in different experiments did not significantly differ in prime visibility [*F*(3, 56) = 0.2, *p* > 0.05]. The finding indicated that even after training, the primes were still not consciously discernible to the participants. In order to better explain the relationship between prime visibility and the priming effect in this study, the difference in prime visibility test and free choice congruent response rate between pre and post training was analyzed using Pearson Correlation Analysis. The results showed that the correlation coefficient was 0.05 and the *p* value was 0.70. The results showed that there was no significant correlation between the changes of prime visibility and free choice congruent response rate from before to after training.

The reaction times were analyzed using a 2 × 3 repeated-measures ANOVA with before and after training (pre-test and post-test) and prime-response congruencies (congruent, incongruent and neutral) as factors. In Experiment 1, a Greenhouse–Geisser correction was used for tests involving the factor prime-response congruency, which violated the ANOVA assumption of sphericity. This analysis yielded a significant main effect of pre-and post-training [*F*(1, 14) = 21.87, *p* < 0.001] and prime-response congruency [*F*(1.18,16.50) = 33.20, *p* < 0.001]. The significant main effects of pre-and post-training indicated that the responses in the post-test were faster than those in the pre-test (pre-test minus post-test = 45 ms). The results showed a significant congruency effect such that the prime-congruent responses were significantly faster (*p* < 0.001) than the prime-incongruent responses (incongruent–congruent = 25 ms). Pairwise comparisons employing Bonferroni corrections revealed that directional primes led to a significant facilitation effect such that the prime-congruent responses were faster than the prime-neutral responses (neutral–congruent = 9 ms), and a significant interference effect was observed such that the prime-incongruent responses were slower than the prime-neutral responses (incongruent–neutral = 17 ms). The interaction between pre/post-training and prime-response congruency was not significant [*F*(2, 28) = 0.56, *p* > 0.05], indicating that the two variables of pre/post-training and prime-response congruency were independent. The reaction time results of Experiments 2, 3 and 4 also had the same tendency (see Table 1 for specific data). These findings showed that a stable subliminal priming effect could be produced under the four experimental conditions, and the reaction speed was improved to a certain extent following training.

**Table 1:** Reaction time in free choice trials in four experiments.

	Reaction time (ms)		
	Congruent	Incongruent	Neutral
Pre-test			
Exp. 1	427.6 (15.9)	454.6 (14.6)	434.6 (15.0)
Exp. 2	471.4 (19.9)	505.2 (22.5)	485.3 (21.9)
Exp. 3	442.8 (10.3)	481.1 (9.9)	455.4 (9.4)
Exp. 4	450.2 (19.5)	478.1 (16.5)	457.6 (18.5)
Post-test			
Exp. 1	382.7 (11.4)	406.2 (10.9)	392.8 (19.4)
Exp. 2	415.6 (20.5)	438.4 (20.5)	423.4 (18.2)
Exp. 3	418.5 (11.6)	449.4 (11.2)	431.6 (12.3)
Exp. 4	389.6 (14.6)	417.4 (13.7)	397.0 (13.1)

Numbers in parentheses show the standard errors of the means.

**Figure 4:**
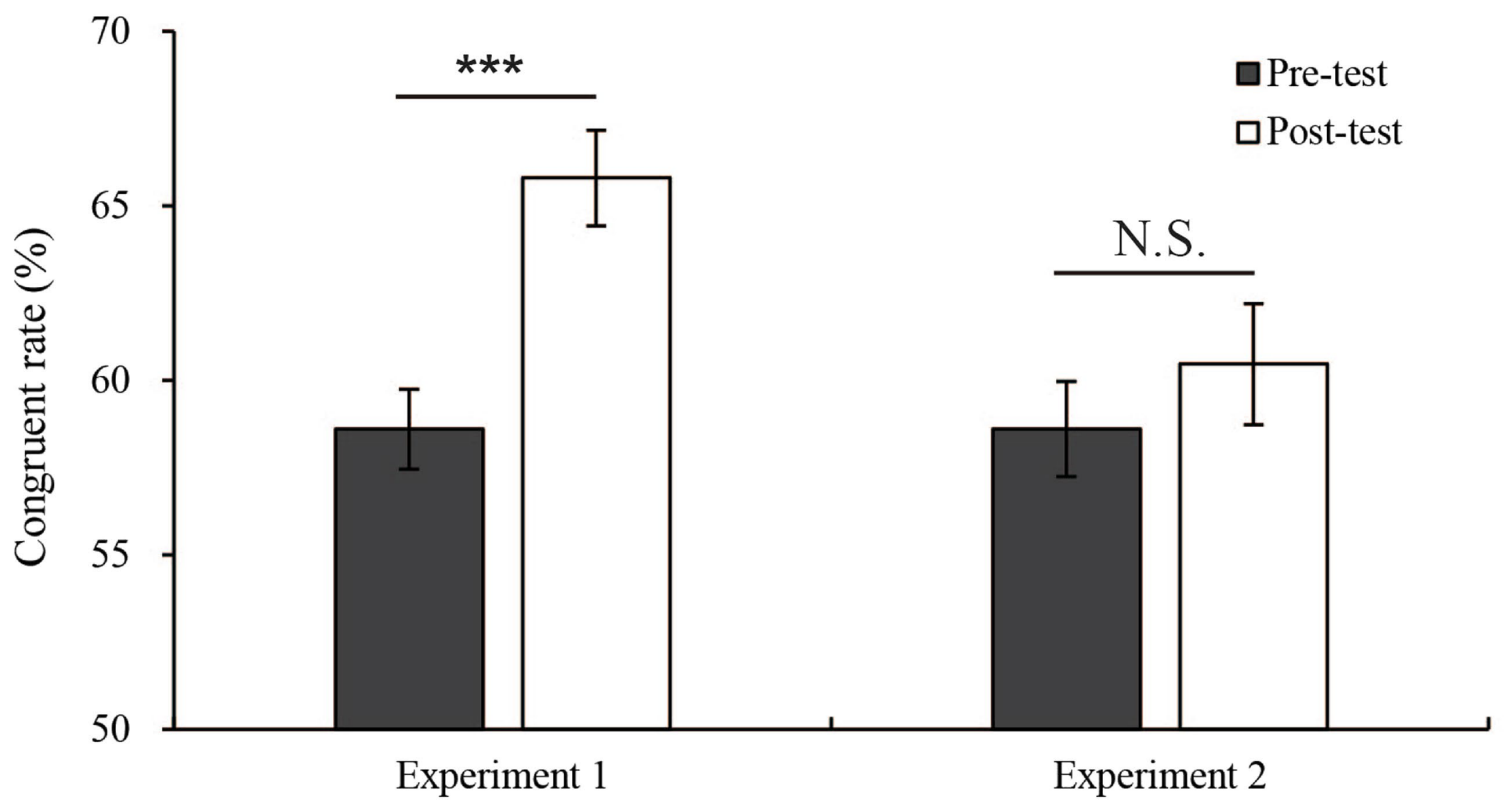
Mean congruent response rates in the pre-test and post-test free-choice trials in Experiments 1 and 2. Error bars show the standard error of mean. ^***^p < 0.001. ^**^p < 0.01. ^*^p < 0.05. N.S. indicates a nonsignificant difference.

To evaluate whether subliminal learning enhanced the masked priming effect, the free-choice congruent response rate was analyzed using a 2 (pre-test vs. post-test) × 2 (group: Experiment 1 vs. Experiment 2) mixed analysis of variance. There was a significant main effect of pre/post-training [*F*(1,28) = 19.18, *p* < 0.001, *η*^2^ = 0.41], but the main effect of groups was not significant (*p* > 0.05). Pairwise comparisons employing Bonferroni corrections for both group variable and pre-training/post-training conditions revealed that the post-test congruent response rate was higher than the pre-test congruent response rate (post-test vs. pre-test p < 0.001), but there was no significant difference between the group variable. Additionally, there was a significant interaction between pre-training/post-training and groups [*F*(1,28) = 6.64, *p* = 0.016, *η*^2^ = 0.19]. The simple effect test showed that in Experiment 1, the simple effect of the pre-test/post-test variable was significant [*F*(1,28) = 24.19, *p* < 0.001, *η*^2^ = 0.46], while in Experiment 2, i.e., the control group, the simple effect of the pre-test/post-test variable was not significant (*p* > 0.05). In the pre-test, the simple effect of the group variable (Experiment 1 vs. Experiment 2) was not significant (*p* > 0.05), while in the post-test, the simple effect of the group variable was significant [*F*(1,28) = 5.83, *p* = 0.023, *η*^2^ = 0.17]. Therefore, the difference between the results obtained before and after training was caused by the group difference (see Figure 4). Compared with the learning effect in Experiment 2, the learning effect in Experiment 1 was stronger. In the forced choice trials, to evaluate whether subliminal learning enhanced the masked priming effect in forced choice trails, the forced choice error rate difference (incongruent error rate minus congruent error rate) was analyzed using a 2 (pre-test vs. post-test) × 2 (group: Experiment 1 vs. Experiment 2) mixed analysis of variance. There was a significant main effect of pre-post training [*F*(1,28) = 5.14, *p* < 0.05, *η*^2^ = 0.16], and the main effect of groups was not significant (*p* > 0.05). Pairwise comparisons employing Bonferroni corrections for both group variable and pre-training/post-training conditions revealed that the post-test forced choice error rate difference was higher than the pre-test forced choice error rate difference (post-test vs. pre-test *p* < 0.05), but there was no significant difference between the group variable. Additionally, there was no significant interaction between pre-post training and groups (*p* > 0.05).

**Figure 5:**
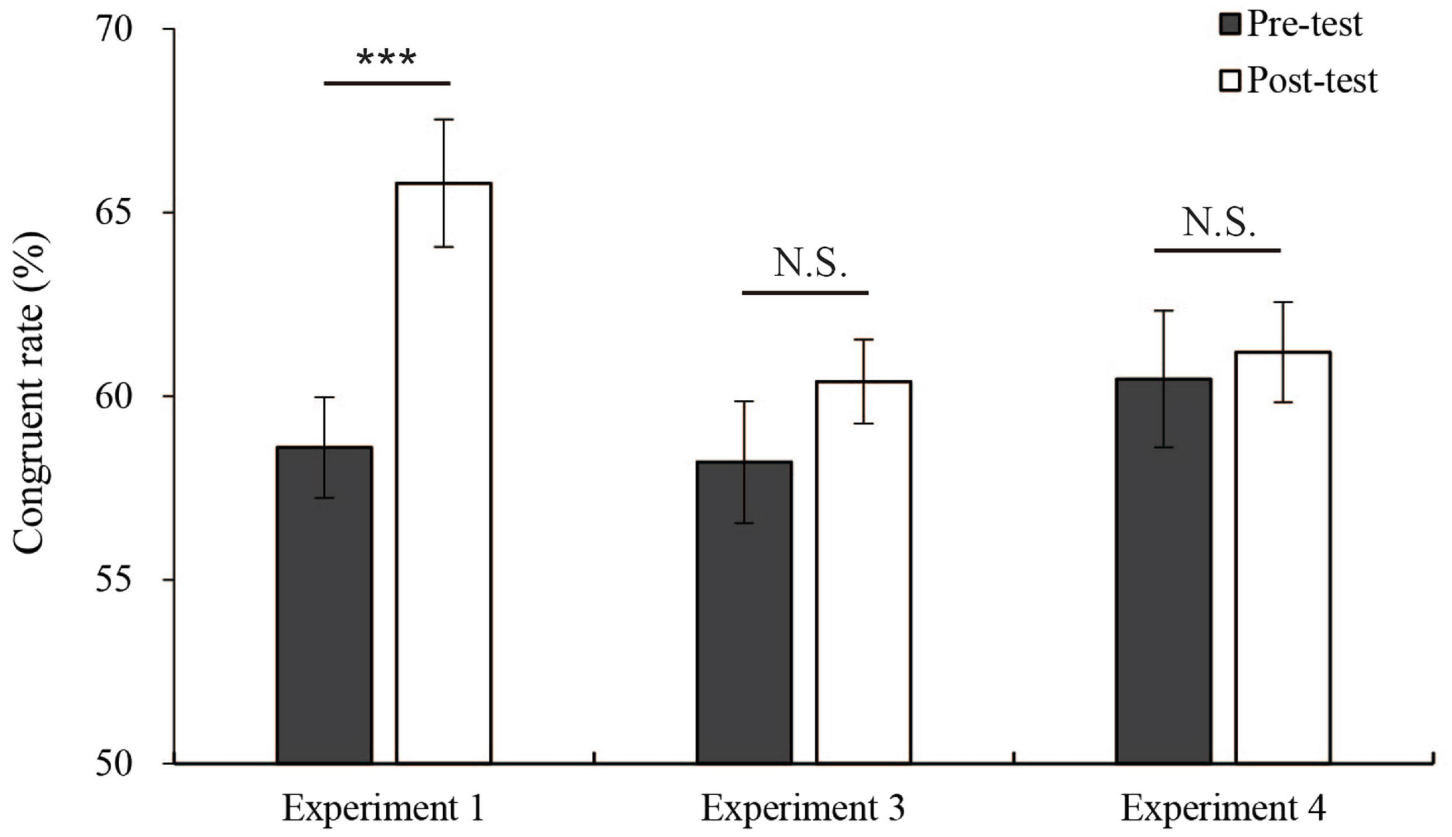
Mean congruent response rates in the pre-test and post-test free choice trials in Experiments 1, 3 and 4. Error bars show the standard errors of mean. ^***^p < 0.001. ^**^p < 0.01. ^*^p < 0.05. N.S. indicates a nonsignificant difference.

To evaluate whether the ratios of free choice to forced choice trials during training had an impact on the learning effect, the free choice congruent response rate was analyzed using a 2 (pre-test vs. post-test) × 3 (groups: Experiments 1 vs. 3 vs. 4) mixed analysis of variance. There was a significant main effect of pre-post training [*F*(1,42) = 13.96, *p* < 0.001, *η*^2^ = 0.25], and the main effect of groups was not significant (*p* > 0.05). Pairwise comparisons employing Bonferroni corrections for both group variable and pre-training/post-training conditions revealed that the post-test congruent response rate was higher than the pre-test congruent response rate (post-test vs. pre-test *p* < 0.01), but there was no significant difference between the group variable. Additionally, there was a significant interaction between pre-post training and groups [*F*(2,42) = 4.77, *p* = 0.014, *η*^2^ = 0.19]. The simple effect test showed that in Experiment 1, the simple effect of the pre/post-test variables was significant [*F*(1,42) = 21.31, *p* < 0.001, *η*^2^ = 0.34], while in Experiment 3 and Experiment 4, the simple effect of pre/post-test variables was not significant (Experiments 3 and 4: *p* > 0.05). Therefore, the difference between the results obtained before and after training was affected by the group difference (see Figure 5). In addition, the forced choice error rate difference was also analyzed used a 2 (pre-test vs. post-test) × 3 (groups: Experiment 1 vs. 3 vs. 4) mixed ANOVA. There was a significant interaction between pre-training and group [*F*(2,42) = 4.77, *p* = 0.039, *η*^2^ = 0.14]. The simple effect test showed that in Experiment 1, the simple effect of the pre-/post-test variable was significant [*F*(1,42) = 9.73, *p* = 0.003, *η*^2^ = 0.19], while in Experiments 3 and 4, Simple effects of pre-/post-test variables were not significant (Experiments 3 and 4: *p* > 0.05).Compared with Experiments 3 and 4, the learning effect in Experiment 1 was significant. This result showed that when the proportion of free choice and forced choice trials are equal, the increase caused by repeated practice was stronger than that under the condition of unequal proportions. This finding was consistent with the situation that we mentioned in the introduction. Switch trials have a stronger effect on the free choice priming effect than repeat trials. We discuss this result in more detail in the “Discussion.”

To evaluate how the learning effect varied across training sessions (Sessions 1–4) in the training phase, a one-way within-subjects repeated measures ANOVA was conducted to compare the effect of number of training sessions (Session 1 vs. 2 vs. 3 vs. 4) on free choice congruent response rate in the training. In Experiment 1 (free-choice: forced-choice = 50:50), there was a significant main effect of training times [*F*(3,42) = 8.04, *p* < 0.001, *η*^2^ = 0.37]. Pairwise comparisons employing LSD corrections revealed that the congruent response rates in Sessions 4 (*M* = 64.4% SD = 4.1%) were significantly higher compared with the Session 1 (*M* = 58.8% SD = 4.5%) and Session 2 (*M* = 59.7% SD = 4.3%) congruent response rate (*p* < 0.001 and *p* < 0.01, respectively), the congruent response rates in Sessions 3 (*M* = 62.4% SD = 5.9%) were also significantly higher compared with the Session 1 and Session 2 congruent response rate (*p* = 0.040 and *p* = 0.037, respectively; see figure). However, in Experiment 3 (free-choice: forced-choice = 10:90), there was not a significant main effect of training times (*p* = 0.052, Session 1: *M* = 52.9% SD = 2.0%, Session 2: *M* = 50.2% SD = 2.7%, Session 3: *M* = 50.3% SD = 2.5%, Session 4: *M* = 54.7% SD = 2.0%). In Experiment 4 (free-choice: forced-choice = 90:10), and there was also not a significant main effect of training times (*p* = 0.810). Pairwise comparisons using LSD correction also did not find any significant differences from session to session in Experiment 3 and Experiment 4. These results showed that in the training phase, the learning effect of Experiment 1 was significant compared to Experiments 3 and 4. This shows that in Experiment 3 and Experiment 4, the reason why the congruent response rate of the post-test was not higher than pre-test due to training is not because the ratio of the training task to the test-task has changed, but because the trial type proportions themselves could not produce learning effects.

**Figure 6:**
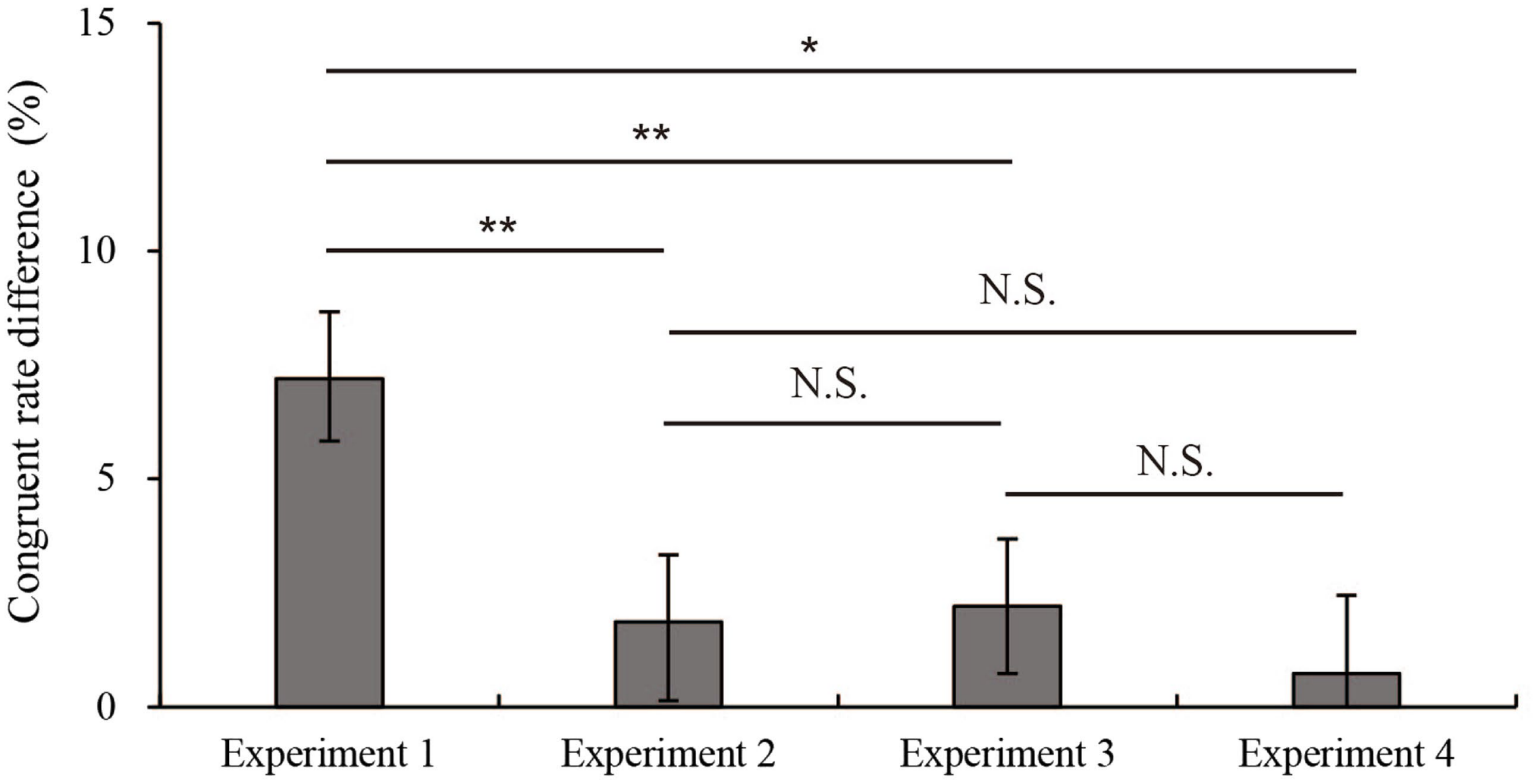
Mean differences in the congruence rates between the pre-test and post-test in Experiments 1–4. Error bars show the standard errors of mean. ^***^p < 0.001. ^**^p < 0.01. ^*^p < 0.05. N.S. indicates a nonsignificant difference.

Figure 6 shows the results of a one-way ANOVA of the difference between the pre-and post-test results. The difference obtained by subtracting the congruent response rate in the pre-test from the congruent response rate in the post-test of the four experiments as the dependent variable was analyzed using a one-way between-subjects ANOVA. There was a significant effect at the *p* < 0.05 level in the difference between the pre-and post-test results [*F*(3, 56) = 3.49, *p* = 0.021]. The *post hoc* comparisons performed by using the LSD test indicated that the mean difference in Experiment 1 (*M* = 7.2%, SE = 1.5%) was significantly higher than those in Experiment 2 (*M* = 1.9% SE = 1.5%), Experiment 3 (*M* = 2.2%, SE = 1.5%) and Experiment 4 (*M* = 0.7%, SE = 1.5%). However, Experiment 2 (control group) did not significantly differ from Experiments 3 and 4. Taken together, these results suggested that only Experiment 1 produced a significant increase from the pre-test and post-test.

## Discussion

The present study mainly had two aims. The first aim was to explore the role of subliminal learning in free choice. The results of Experiment 1 showed that the subliminal priming effect in free choice could be increased. However, in Experiment 2 (control group), the congruent response rate in the post-test was not significantly higher than the congruent response rate in the pre-test. Second, this study used three experimental conditions with different ratios of free choice to forced choice trials in a training block, which represented higher switch trials and higher repeat trials, respectively. We compared the learning effect and wondered which of the two had a greater impact on the priming effect. We observed that the subliminal learning effect in Experiment 1 (50% free choice trials) was significantly higher than that in Experiment 3 (10% free choice trials) and Experiment 4 (90% free choice trials). In all four experiments, the prime visibility test results were not significantly changed by practice. These results suggested that the effect of free choice masked priming was enhanced by repeated practice with equal proportions of free choice and forced choice trials, i.e., higher switch trials.

According to the results of the present study (see Figure 4), repeated practice can enhance the intensity of masked priming on free choice. In this study, the participants’ congruent response rate in Experiment 1 significantly increased from before to after training. This finding is probably related to the metacontrast masking paradigm. Metacontrast masking refers to the suppression of the visibility of a briefly flashed target stimulus by a similarly brief and spatially adjacent mask stimulus that follows the target in time at varying stimulus onset asynchronies (SOAs; Breitmeyer et al., 2008). Some neurophysiological and psychophysical evidence shows that within the perception system, processing is interrupted by metacontrast masks, and targets remain invisible if new input arrives before boundary contours have been computed and filled in (Bridgeman, 1988; Francis, 1997; Breitmeyer and Ogmen, 2000). The brain automatically temporarily discards prime information. Instead, they process target information and making choices according to target information. During the decision-making response process, people’s cognitive resources collect and evaluate all types of information from as much time as possible and respond according to this information, linking arbitrary sensory output to action (Vorberg et al., 2003). If the prime information is relevant to the decision at this time (whether external shape or semantic information), the decision-making process assigns it a certain weight; thus, the decision is influenced by a subliminal prime to produce a priming effect (Bermeitinger and Hackländer, 2018). In our study, we found that the practice trials increased the rate and frequency of the subliminal priming effect and made it easier to produce this phenomenon; thus, both the reaction speed and the congruent response rate increased. In Experiment 2, i.e., the control group, the prime stimulus included no directional information during training, and there was no process to bias free choice. Thus, there was no learning effect. Furthermore, the presentation position of the stimulus during the training stage and the test stage were different, indicating that the learning effect in Experiment 1 does not only stay in the trained spatial position, but extends to the untrained spatial position. This learning effect can be generalized to untrained spatial locations and stimulus pointing directions. Law and Gold used trained monkeys to detect the direction of a moving point. The authors also used recording electrodes to record the potential changes of MT and LIP and found that behavioral performance enhancement was accompanied by changes in the motor signal of the LIP rather than the MT (Law and Gold, 2008). Correspondingly, many studies have also shown that perceptual learning can create changes in higher-level, nonvisual areas involved in information accumulation and integration (Fahle, 2005; Law and Gold, 2009; C. T. Law and Gold, 2010). This finding is consistent with the prime visibility test’s results of the present study and suggests that increase involved in perceptual learning lies not in the way sensory information is expressed in the brain but how sensory performance is interpreted as a decision to guide behavior. Therefore, the learning effect in this study was likely derived from a significant increase in the ability to integrate and interpret the masked prime.

Previous studies have shown that the processing of unconscious information increase with training, but these increase only manifest when there is decision-relevant conscious information to bind to the unconscious information. That is the masked stimuli seem difficult to use and learn if they are not bound to conscious information (Vlassova et al., 2014; Vlassova and Pearson, 2018). However, in masked priming free choice paradigms, the masked prime is not directly related to free choice target stimuli. The results of this study demonstrate that training with masked priming can also produce subliminal learning effects under free choice conditions in which conscious decision information and masked stimuli are not directly bound. As we mentioned in the introduction section, a study by Schlaghecken and Eimer (2004) showed that only primes that are a part of the currently active task set affect the motor system. When this prime target (S–R) is no longer sufficiently relevant, the effect appears to fail rapidly. These results are important because they show the role of S–R mapping in generating free choice masked priming. In forced choice trials, one target stimulus corresponds to only one response (e.g., see the left arrow and press the left button). Hommel, 1997; Elsner and Hommel (2001) proposed the following hypothesis regarding S–R mapping: the response creates a representation of the action of a particular stimulus that combines the codes of the stimulus properties with the corresponding action codes (stimulus–response). These representations can be stored in memory such that they can be used in subsequent tasks (both free choice and forced choice tasks). Therefore, a stronger stimulus–response association could lead to a stronger free choice masked priming effect. In this study, there was no direct binding relationship between the masked information (prime) and conscious information (free choice target) in the free choice trials. However, it appears in the same task set, and the S-R mapping of the prime and forced choice targets can be shared by free choice, leading to a subliminal learning effect that can be found in free choice masked priming.

In addition, the increase in free choice masked priming can be explained by the diffusion drift model of decisions in two-alternative forced-choice (2AFC). The Diffusion models were developed to account for decisions that involve two choices and require rapid choice (Ratcliff, 1978; Ratcliff and Rouder, 1998). In the diffusion drift model, the drift rate is the average rate at which information accumulates, where higher drift rates lead to faster and more accurate responses. Vlassova and Pearson’s study also suggested that better post-training performance increased drift rate, i.e., more efficient accumulation of subliminal evidence (Vlassova and Pearson, 2018). Similarly, a neuroimaging study has shown that a key brain region involved in free choice is the RCZ, which is a part of the medial frontal cortex. When making a free choice, the activity of the RCZ can be regulated by a masked prime in a bottom-up manner (Teuchies et al., 2016). Such studies note that under a free choice condition, our brains still automatically select and use external information, even if such information is derived from subliminal stimuli. We assume that the ability to extract and select external sensory information is improved after many training sessions. With increases in stimulus–response correlations, participants are more likely to use masked primes to assist in free choice trials, which may explain the presence of the subliminal learning effect in the free choice trials.

In the free choice trials, there is a significant interaction between pre-training/post-training and groups (Experiment 1 vs. Experiment 2), which showed that the increase caused by repeated practice in Experiment 1 was stronger than in Experiment 2 (control group). This result suggested that Experiment 1 training was more effective than the control experiment in the free choice trials. However, this interaction was not observed in forced-choice trials. We speculate this is due to the different response intentions for free choice and forced choice. In a study by Naefgen et al. (2018), the authors found the reaction time of free choice trials is always longer than forced choice trials. They propose that response choice in both tasks relies on information accumulation toward a specific goal. While in forced choice tasks, this goal is externally determined by the stimulus, in free choice tasks, it needs to be generated internally, which requires additional time. Additionally, in the free choice trials, priming is based on the integration of external stimulation by the prime and internal response tendencies, the internal response tendencies are more likely to be influenced by masked information (Mattler and Palmer, 2012). It means that free choice tasks are more likely to be influenced by subliminal information than forced choice tasks. Therefore, the present study suggested that the difference between free and forced choice response intentions leads to the fact that training in forced choice trials is less effective than in free choice trials.

As shown in Figure 5, only Experiment 1 (50% free choice trials) had a significant learning effect, but no significant learning effect was found in Experiment 3 (10% free choice trials) or Experiment 4 (90% free choice trials). And according to congruent response rate results in the training phase, only in the Experiment 1 training phase, as the number of training sessions increased, did the masked priming effect become stronger. However, no significant enhancement effect was found in Experiment 3 and 4. This result indicates that the lack of learning effect in Experiment 3 and 4 is not due to the change of procedure from training to test. Based on the assumptions made in the introduction, it can be assumed that switch trials between free choice and forced choice trials are more likely to affect the free choice masked priming effect than repeat trials. A previous study has shown that endogenous and exogenous action plans perform similarly and, therefore, are not controlled by separate endogenous and exogenous systems (Richardson et al., 2020). In Experiment 1 of this study, when the proportion of free choice and forced choice trials was the same, the switch times between the free choice and forced choice trials were the highest. Thus, in this case, the content from the forced choices S–R mapping can be better shared with the representation of the free choice task. Free choices can more effectively invoke the content of S-R mapping. Pfister et al. also found that when free choice and forced choice trials were randomly mixed, the participants were more likely to use an intention-based mode of action control that was more similar to free choice (Pfister et al., 2010), which could facilitate the sharing of representation of S-R mapping content. A study also showed that when participants switch between different tasks, they are more likely to adopt flexible response strategies and are more susceptible to being affected (Dreisbach and Fröber, 2019). However, under the 10% or 90% free choice conditions (Experiment 3 and Experiment 4, respectively), this type of switching occurred less often, and it was difficult to help the free choice case obtain the S–R mapping relationship. Likewise, in a study by Wenke et al. (2010), the authors calculated the congruent response rate in priming effects with ratios of 25/75 and 75/25 free choice to forced choice trials. The interaction between the ratio and congruent response rate was not significant, indicating that the congruent response rate did not depend on the frequency of forced choice and free choice trials within a block. Especially in experiment 3, although the forced choice was repeated many times, S-R mapping should be increased (Gozli, 2019). For the above reasons, we speculate that when the proportion of free choice to forced choice trials is the same, the S-R mapping of forced choice trials can be better transferred to free choice trials, rendering it easier to use masked primes to assist free choice reactions than when the proportions of free choice to forced choice trials are unequal. Another possible explanation is that the effect is a transfer appropriate processing effect (Morris et al., 1977; Bransford and Schwartz, 1999). That is, if the test matches the way how learning is done, the transfer from learning to testing would occur. We changed the stimulus presentation location, stimulus orientation, and response mode from the training to the testing phases. In this way, a transfer appropriate processing interpretation was avoided as much as possible. However, we cannot completely rule out a possible effect of transfer appropriate processing on the experimental results. It is also a topic worthy of future research. In addition, this study used a between-subject design as a training experiment. This means that it is difficult to completely exclude individual differences between groups, and how to exclude the influence of individual differences in future research is also a topic worth exploring.

## Conclusion

Overall, the data showed that the free choice process was influenced by masked primes *via* subliminal learning. This study found that when the proportions of free choice and forced choice trials in a block were the same, the subliminal learning effect on free choice was the strongest. This study indicated that compared to repeat trials, switch trials between free choice and forced choice trials had a stronger priming effect on free choice responses. In addition, this study indicates that the masked priming effect could be enhanced by improving the participants’ conceptual understanding of the stimulus–response mapping correlation. In conclusion, these findings indicate that subliminal learning can effectively accumulate and integrate masked primes, thereby making better use of available subliminal stimuli to affect free choice responses.

## Data availability statement

The raw data supporting the conclusions of this article will be made available by the authors, without undue reservation.

## Ethics statement

The studies involving human participants were reviewed and approved by The Ethics Committee of Okayama University. The patients/participants provided their written informed consent to participate in this study.

## Author contributions

QD, LY, and QW conceived and designed the experiments. YY made the program. QD, LY, and WL collected the data, analysed the data. QD and LY wrote the draft manuscript, and received comments from QW, JY, ST, YE, and JW. All authors contributed to the article and approved the submitted version.

## Funding

This research was supported by the Japan Society for the Promotion of Science (JSPS) Kakenhi (grant numbers 19KK0099, 20K04381, 20K07722 and 22K04011) and a Grant-in-Aid for Strategic Research Promotion from Okayama University.

## Conflict of interest

The authors declare that the research was conducted in the absence of any commercial or financial relationships that could be construed as a potential conflict of interest.

## Publisher’s note

All claims expressed in this article are solely those of the authors and do not necessarily represent those of their affiliated organizations, or those of the publisher, the editors and the reviewers. Any product that may be evaluated in this article, or claim that may be made by its manufacturer, is not guaranteed or endorsed by the publisher.
